# The Hummingbird Project year 2: decreasing distress and fostering flourishing in a pragmatic pre–post study

**DOI:** 10.3389/fpsyg.2024.1257446

**Published:** 2024-03-25

**Authors:** Ian Andrew Platt, Kevin D. Hochard, Michelle Tytherleigh, Chathurika Kannangara, Jerome Carson, Claudine McFaul, Catherine North

**Affiliations:** ^1^MedEquip4Kids, Manchester, United Kingdom; ^2^School of Psychology, University of Chester, Chester, United Kingdom; ^3^School of Education and Psychology, University of Bolton, Bolton, United Kingdom; ^4^School of Psychology and Counselling, The Open University, Walton Hall, Milton Keynes, United Kingdom

**Keywords:** school, well-being, positive psychology, child, adolescent, intervention

## Abstract

Multi-component Positive Psychology Interventions (mPPIs) in secondary schools have been shown to improve mental health outcomes for young people. The Hummingbird Project mPPI is a six-week program of workshops designed to introduce a variety of positive psychology (PP) concepts to secondary school-aged children in schools to improve well-being, resilience, and hope. The effects on mental distress, however, were not explored. The current study, therefore, was designed to replicate the effects of the Hummingbird Project mPPI on positive mental health and to also explore the effects on symptoms of mental distress. Secondary school-aged children (*N* = 614; mean age = 11.46 years) from a sample of secondary schools located across the North West of England (*N* = 7) participated in the study; the majority of children were in Year 7 (94%). The PP concepts explored included happiness, hope, resilience, mindfulness, character strengths, growth mindset, and gratitude. The results showed significant improvements associated with the mPPI in well-being (as measured by the World Health Organization Well-Being Index; WHO-5), hope (as measured by the Children’s Hope Scale; CHS), and symptoms of mental distress (as measured by the Young Person’s Clinical Outcomes in Routine Evaluation; YP-CORE) from pre- to post-intervention. While acknowledging the limits due to pragmatic concerns regarding the implementation of a control group, the effectiveness of the Hummingbird Project mPPI on well-being was replicated alongside reducing the symptoms of mental distress. Future evaluation, however, will need to implement more robust designs and consider follow-up duration to assess the longer-term effects of the Hummingbird Project mPPI.

## Introduction

Mental health is associated with improvements in educational attainment, greater productivity, reductions in mortality, increases in social interaction and engagement, reduced risk of both suicide and mental illness, reduced likelihood of risk-taking, and increased levels of resilience (Campion et al., 2012). Indeed, mental health is on a spectrum, distinguishing those who flourish and live a good life and function well at the positive extreme, from those who languish, have low levels of satisfaction and may be living a life without purpose, meaning and fulfillment, etc., at the negative extreme (Keyes and Haidt, 2010). Mental illness is the second largest source of burden of disease in England. Mental illnesses are also more common, long-lasting, and impactful than other health conditions (Public Health England, 2019). Notably, 50% of lifetime mental illness starts by age 14 and 75% by the mid-20s (Kessler et al., 2007). In the United Kingdom (UK), 13.6% of children aged 11–16 years can have a mental health disorder at any one time (RCPCH, 2020).

The effects of mental illness in younger life can also be long-lasting. A longitudinal study involving 17,634 children in England, Scotland, and Wales showed an association between psychological problems in childhood and employability in later life (Goodman et al., 2011). The Children’s Society (2020) also showed that, at 15 years of age, children in the UK ranked lowest out of 24 European countries for life satisfaction and having a positive sense of purpose in life. They also ranked second in terms of sadness (The Children’s Society, 2020). Taken together, therefore, understanding why this is and finding ways to reduce mental illness in young people in the UK is a priority.

### The role of schools in young people’s mental health

Globally, children in OECD countries spend an average of over 800 h a year in school (OECD, 2020). Since children spend such a large amount of their time in schools and over several critical periods in their early development, schools have a large role to play in their cognitive, emotional and social development, social skills, academic attainment, and, ultimately, well-being (Fazel et al., 2014). Even so, in the UK, the Department for Education saw a real-term budget cut of 7.4% between 2010 and 2016 (Crawford and Keynes, 2015), which has triggered some significant impacts and challenges in schools. These include staff cuts and poorer retention, scaled-back curriculums, and increased levels of teacher ill-being.

While initiatives for embedding well-being into the curriculum in UK schools are nothing new (e.g., the *Children and Social Work Act 2017* made PSHE [personal, social, health, and economic education] a statutory requirement at all schools in England from September 2020), many tend to run separate well-being initiatives alongside mainstream teaching (Spratt et al., 2006). Mental health outcomes can also be improved by delivering both mental health identification and prevention programs in schools (Levitt et al., 2007). However, the engagement of affected parties, including school staff, counselors, and support staff, is necessary for the successful implementation of evidence-based interventions in school settings, and it has been shown that such engagement is lacking (Fazel et al., 2014). Fazel et al. also identified a number of other challenges associated with the delivery of mental health interventions in schools. These operate on three levels: individual factors, such as stigma, individual risk factors, and parental issues; community-related factors, such as geographic location and social status; and system-related factors, including access to funding, waiting times, and availability of training.

### The role of positive psychology in schools

Positive psychology (PP) and its application in schools through Positive Education provides a more holistic approach to education that addresses both academic success and well-being without one having to overshadow the other. Building on the science of well-being (Seligman, 2002), and more often based on Seligman’s (2011) PERMA Framework (positive emotions, engagement, positive relationships, meaning, and accomplishment) and its’ adaptations, Positive Psychology Interventions (PPIs) are now being developed for schools (Slade et al., 2017; Rashid and Seligman, 2018), and it has been shown that embedding well-being lessons as part of the school curriculum improves student mental health (Boniwell et al., 2016). As a consequence, this includes benefits on academic performance, too. PPIs that run year-round as part of class timetables have also shown efficacy in not only improving well-being but also reducing stress and anxiety (Shoshani and Steinmetz, 2014). Such an approach, however, requires support on a number of levels, including embedding well-being into teacher training, school leadership training, educational culture, and shifting mindsets too (Waters, 2011). To be effective at embedding well-being for students, teachers need to have positive well-being, too.

An alternative approach is to offer brief PPIs that run outside of the school’s usual curriculum. While the results of these have been mixed (Suldo et al., 2014), leading to the conclusion that the widespread adoption of brief school-based PPIs is not empirically supported (Dawood, 2013), more recent evidence is much more optimistic. For example, brief PPIs have been shown to enhance academic performance by increasing students’ motivation to study (Muro et al., 2018). They have also demonstrated a diverse range of benefits to students in terms of both well-being and learning (Shankland and Rosset, 2017). A limitation of the majority of brief PPIs, however, is that they can focus on a single concept from PP, such as gratitude (Froh et al., 2010), character strengths (Quinlan et al., 2015), or mindfulness (Sapthiang et al., 2019).

Although peer-reviewed research in this area is limited, especially when compared to that which uses a single PPI approach, PPIs that broaden the focus to include multiple concepts from positive psychology [Multi-component Positive Psychology Interventions; mPPIs] in schools have been shown to be more effective. For example, in her review of 12 school-based PPIs designed to increase student well-being and academic performance, Waters (2011) concluded that, while acknowledging that some limitations in, e.g., design exist, the effectiveness of PPIs is encouraging. The PP concepts utilized in her review included positive emotions, resilience, and character strengths. In more recent exploratory research of a mPPI designed to improve happiness and classroom behavior in a sample of at-risk high school students, DeBiase et al. (2022) also found that, while the effects were not as expected, even the small changes in a positive direction were promising, and especially for those working with at-risk populations of adolescents. Although limited by the small number of systematic literature reviews that have been carried out on the effectiveness of PPIs, the reporting of smaller effect sizes appears common (White et al., 2019). Indeed, in their meta-analysis of the effectiveness of PPIs more generally (i.e., not just in schools), White et al. found the effect sizes reported were much smaller, although they also justified this by differences across studies and their design, including issues related to methodology and the use of smaller sample sizes.

### Multi-component positive psychology interventions

Given there is no ‘one fits all’ approach to PPIs, and with some PPIs found to be more effective and engaging for some students than others, additional benefits of adopting a mPPI over a single PPI approach include having a broader reach, greater inclusivity and, ultimately, a larger range of beneficial effects. One such mPPI carried out in secondary schools in the UK is the Hummingbird Project (Platt et al., 2020). Drawing on Seligman’s (2011) PERMA Framework, the Hummingbird Project mPPI involves teaching students concepts from positive psychology in class, which they then practice at home, using at-home activities. The delivery of the mPPI is approximately 1 h per week over 5–6 weeks. Indeed, studies discussed above have demonstrated the efficacy of interventions that target each of the individual variables targeted in the Hummingbird Project.

The results of the Hummingbird Project mPPI following its’ first year of academic delivery showed improvements in well-being, resilience, and hope in secondary school-aged children, thus evidencing an increase in flourishing as conceptualized by Keyes and Haidt (2010). A limitation of this study, however, and one which this current study was designed to address, is that the effects on symptoms of mental distress were not also explored. Acknowledging that repeat doses may be necessary (Stockings et al., 2016), previous research has shown that school-based mental health interventions can lead to reliable and clinically significant changes in anxiety and depression (Punukollu et al., 2020). Such information would be of particular interest to schools since mental ill-health has higher costs for schools than other public service sectors (Fazel et al., 2014). It would also be of interest to researchers, as evidence for the effects of school-based anxiety prevention is mixed (Waldron et al., 2018).

### The current study

The current study aimed to explore the effects of the mPPI Hummingbird Project on well-being in secondary school students following its’ second full academic year of delivery. The project had previously been piloted with 90 students in two schools and then delivered to 1,058 students in 14 schools across the North West of England [see Platt et al. (2020)]. Furthermore, it aimed to expand on prior findings of the Hummingbird Project mPPI (Platt et al., 2020), which found a small beneficial effect on indicators of flourishing, by also assessing the impact of the mPPI on symptoms of mental distress.

Given the time, funding, and resource difficulties faced by schools, a full randomized controlled trial was impractical; hence, a pragmatic approach using within-subject comparisons was adopted. The delivery was carried out by a single researcher, assisted by Undergraduate and master’s level psychology students recruited from two universities local to the schools. All participating schools were based in the North West of England and selected from those who chose to respond favorably to the offer of this free mPPI; some variability across schools did exist. As such, the current study explored whether any effect of the mPPI found would remain having accounted for individual, school, or local authority-related differences. These three levels have been chosen as proxies for the levels of individual, community, and system suggested by Fazel et al. (2014) discussed above, each of which is expected to bring unique challenges that may negatively effect the outcomes of the PPI. [note: there are 353 local authorities in England. These provide the local government for their metropolitan borough, county, or district (National Audit Office, 2017)].

Using standardized measures of well-being and mental distress and building on the effectiveness of previous research using the Hummingbird Project (Platt et al., 2020), it was hypothesized that:

- The mPPI would improve the students’ levels of well-being and hope from pre- to post-intervention, having accounted for the potential random effects from local authorities, schools, or participants.
- The mPPI would reduce students’ symptoms of mental distress from pre- to post-intervention, having accounted for the potential random effects from local authorities, schools, or participants.

## Method

### Intervention

Year 2 of the Hummingbird Project involved six weekly 1-h sessions that took place during normal timetabled school hours, at different stages across the academic year depending on timetable availability in each school, delivered by a single researcher (Author 1), with support from Undergraduate and Masters Psychology students (trained by Author 1). An introduction to the mPPI for students and teachers took place in session 1, along with a discussion of what happiness means to each individual in the group. A range of concepts from positive psychology was then discussed in sessions 2 to 5, with activities relating to each concept. The concepts covered in these sessions were as follows: happiness, hope and resilience, mindfulness, character strengths, growth mindsets, and gratitude, respectively. During each session, students took part in a range of activities intended to help them understand and use these concepts in their daily lives and were given a homework task to help them understand how each concept can improve their own mental health. The final session involved a recap of the topics covered in previous sessions, along with advice for students on how to incorporate these concepts into their lives in future.

Delivery of the project took place across the academic year in eight secondary schools across North West England. However, delivery of the project had to be halted at one school before post-intervention testing could take place due to the imposition of lockdowns in relation to the coronavirus pandemic. All students were given a workbook to complete throughout the intervention, which then became theirs to keep at the end. The workbook was a place where students could record any work they completed in sessions and at home, as well as their thoughts and feelings regarding the experience. Students were also given questionnaires to complete as baseline and outcome measures.

### Participants

Participants were an opportunity sample of 727 students. However, due to COVID-19 restrictions, complete data could not be obtained from 113 students in one school. As such, our final sample consisted of 614 students. Recruitment of participants involved emailing the Head Teachers and Special Educational Needs Coordinators of a number of schools in the region. Schools in the Cheshire West and Chester Local Authority were selected based on their location being in the top 40% for deprivation (MHCLG, 2019). The participant inclusion criteria for the intervention, which were provided by the research team to the school, were as follows: (a) studying at a secondary school and (b) willingness of the school, parents, and/or carers for students to take part in the study. Furthermore, school staff allowed students to participate based on the student’s form group, PSHE group, timetabled lesson group, or perceived need for intervention; the researchers were not privy to the reason for perceived need. The schools were in control of this process, without interference from the research team.

Participants were aged between 11 and 15 (*M* = 11.46, *SD* = 0.70), and 46% were female, which was fairly representative of all schools in the study. A majority of participants (94%) were in Year 7, 2% were in Year 8, 2% were in Year 9, and 3% were in Year 10. White British students made up 82% of the sample, with the next most populous ethnic group being British Asian, at 8%, students of a mixed background making up 5%, and Black British being 4% of the sample. The remainder were of Polish, Romanian, Turkish, and Hungarian descent.

### Instruments

Questionnaires included three standardized quantitative measures. These were as follows:

- The World Health Organization-Five Well-Being Index (WHO-5; Staehr, 1998) is a five-item well-being scale, with items measured on a 6-point Likert scale (0 = at no time; 5 = all of the time). Staehr (1998) has shown that Cronbach’s *α* = 0.75, and an example item is “I have felt cheerful and in good spirits.” The WHO-5 has been validated as a measure of well-being for adolescents (De Wit et al., 2007), showing a one-factor structure using confirmatory factor analysis. Cronbach’s *α* was 0.82, and the WHO-5 showed a moderate to strong correlation with the CES-D (*r* = 0.67), with the mental health (*r* = 0.60) and self-esteem (*r* = 0.43) subscales of the CHQ-CF87, and with the DFCS (*r* = 0.34), which was taken to confirm concurrent validity. The same study used ROC curve analysis to show the WHO-5 cutoff point of 50 for the identification of mild-to-severe depressive affect, with sensitivity at 89% and specificity at 86%. For our sample, the WHO-5 demonstrated good reliability in both pre-intervention (*α* = 0.73) and post-intervention (*α* = 0.84).
- The Young Person’s Clinical Outcomes in Routine Evaluation (YP-CORE; Twigg et al., 2016) is a monitoring tool with items covering anxiety, depression, trauma, physical problems, functioning, and risk to self. Items are rated on a 5-point Likert scale (0 = not at all; 4 = most or all of the time). An example item is “I have not felt like talking to anyone.” Twigg et al. (2016) showed a one-week test–retest mean Time 1 score of 8.3 (95% CI: 7.2–9.5; range: 0–27; SD = 5.6) and mean Time 2 score of 7.7 (95% CI: 6.5–9.3; range: 0–30; SD = 6.6). Mean change was shown not to be statistically significant (*t* = 1.2, df = 89, *p* = 0.23; Wilcoxon *U* = 1787, *p* = 0.15) with a negligible effect size (Hedges’ *g* = 0.09, 95% CI:-0.21 to +0.39). They found that Pearson’s correlation coefficient for Time 1 and Time 2 scores was as follows: 0.76 (95% CI: 0.65–0.86) and Spearman’s rho was 0.74 (95% CI: 0.58–0.83). They also demonstrated reliability for the YP-CORE when used in a non-clinical sample of children, with Cronbach’s α = 0.83. Similarly, our sample demonstrated good reliability pre-intervention (*α* = 0.81) and post-intervention for (*α* = 0.84) the YP-CORE.
- The Children’s Hope Scale (CHS; Snyder et al., 1997) is a measure of hope that uses six items on a 6-point Likert scale (1 = none of the time; 6 = all of the time). An example item is “I can think of many ways to get the things in life that are most important to me.” Snyder et al. (1997) demonstrated that the CHS has high internal consistency (Cronbach’s Alpha = 0.72–0.86) and high temporal stability [*r* (359) = 0.17, *p* < 0.01]. The internal reliability of the CHS was high for our sample, both pre-intervention (*α* = 0.87) and post-intervention (*α* = 0.92).

### Data collection procedures

Pre-intervention outcome measures were administered to participants at the start of session 1 of the mPPI, with post-intervention measures being administered at the end of session 6. Participants were assured of their anonymity in the outcome testing, encouraged to be as honest as possible, and informed that they had the right to withdraw from the study if they so wished. They were also informed that their school grades would not be affected by their participation in the study or by any of the answers they gave to the questions. Procedures were conducted in line with relevant guidelines and approved by the university ethics board. Once participants had completed both pre- and post-intervention questionnaires, responses were coded, and scores were calculated in line with each instrument’s published scoring guidance.

### Data analysis procedure

The repeated measures outcomes followed a nested structure: within participants (*n* = 614), within schools (*n* = 6), and within local authorities (*n* = 2).

All analyses were performed using the open software Jamovi (version 2.0.0, The Jamovi Project, 2021). To test our hypotheses, mixed-effect model analyses were conducted as these provide more robust estimates than repeated measures ANOVA with respect to missing data and type 1 error rate (Mallinckrodt et al., 2001). Further mixed-effect models account for fixed and random effects, which provide a more comprehensive overview of the effect in the data by accounting for both the overall trends and the individual variations. These analyses were performed in the General Analyses for Linear Models in Jamovi (GAMLj) package version 2.4.8 (Gallucci, 2019). The GAMLj package is able to account for the nested data structure without explicit specification. Three models (one per outcome) were computed, which included the repeated measure (pre and post) as our fixed-factor predictor (time) and included correlated participant level, school-level, and county-level random intercepts, using a restricted maximum-likelihood function with the bobyqa optimizer.

## Results

Descriptive statistics for our full sample for each of the repeated measures outcome variables are presented in Table 1.

**Table 1:** Descriptive statistics for outcome variables.

	Pre		Post	
	Mean	SD	Mean	SD
Wellbeing (WHO-5)	13.40	5.10	14.19	6.02
Mental distress (YP-CORE)	1.31	0.78	1.25	0.82
Hope (CHS)	22.58	7.25	23.25	8.22

WHO-5, World Health Organization-Five well-being index, YP-CORE, young person’s clinical outcomes in routine evaluation, CHS, children’s hope scale.

The mixed-effect models reported displayed approximately normally distributed, homoscedastic residuals. Significant main effects of time were consistently observed across all of the outcome variables (see Table 2), indicating the intervention to have salutary effects. However, the marginal *R*^2^ were low for all models, suggesting a small effect of the fixed factor. ICC for the models showed that the clustering for participants was substantial for all models. However, the similarity of observations by school and county was much less important for the WHO-5 than for our YP-CORE and CHS outcomes. It should be noted that results were found to be unchanged by removing all non-year seven students.

**Table 2:** Mixed-effect models for well-being (WHO-5), mental distress (YP-CORE), and hope (CHS) outcomes.

	Well-being (WHO-5)					Mental distress (YP-CORE)					Hope (CHS)				
		95% CI					95% CI					95% CI			
Fixed effects	Estimates	LL	UL	*t*	*p*	Estimates	LL	UL	*t*	*p*	Estimates	LL	UL	*t*	*p*
Intercept	13.07	11.80	14.34	20.13	0.04	1.46	1.07	1.84	7.39	0.090	21.49	18.39	24.60	13.58	0.052
Time	0.79	0.36	1.21	3.64	< 0.001	−0.08	−0.14	−0.03	−3.14	0.002	0.79	0.30	1.28	3.16	0.002

Random effects (intercept)	*σ* ^2^	ICC	Model fit			*σ* ^2^	ICC	Model fit			*σ* ^2^	ICC	Model fit		
			AIC	*R*^2^ marginal	*R*^2^ conditional			AIC	*R*^2^ marginal	*R*^2^ conditional			AIC	*R*^2^ marginal	*R*^2^ conditional
County	0.48	0.04	6655.50	0.01	0.62	0.06	0.26	2127.45	<0.01	0.75	3.53	0.19	7109.22	<0.01	0.75
School	0.68	0.05				0.04	0.20				3.54	0.19			
Participant	18.03	0.60				0.41	0.71				40.29	0.72			
Residual	11.92					0.17					15.49				

LL and UL, lower limit and upper limit for 95% confidence intervals; *σ*^2^, variance; ICC, intraclass correlation coefficient; AIC, Akaike information criterion; *R*^2^ marginal, variance explained by the fixed effects over the total (expected) variance of the dependent variable; *R*^2^ conditional, variance explained by the fixed and random effects over the total (expected) variance of the dependent variable.

## Discussion

The findings of this study indicate significant positive improvements in all three of the outcome measures, with increases of 0.79 in well-being (WHO-5) and 0.67 in hope (CHS) and very small reductions of −0.08 in symptoms of mental distress (YP-CORE) from pre- to post-intervention, while also accounting for random effects from the local authority, school, and participants. As such, these findings replicate the effects on well-being observed by Platt et al. (2020) following the first year of delivery of the Hummingbird Project mPPI. Like Shoshani and Steinmetz (2014), following a year-round delivery of PPIs, they also show that, even over a much shorter time period of delivery (i.e., five to six weeks), the Hummingbird Project mPPI also reduces mental distress alongside improving well-being. This is further supported by the small marginal *R*^2^ in the models (0.005–0.01). The inter-class correlations (ICCs) for the models also support the inclusion of random intercepts for participants by accounting for a significant amount of variance in scores. This was particularly noticeable in the model of well-being, indicating individual differences among participants to account for some of the effects observed. Conversely, school and local authorities had a small ICC, suggesting that these did not account for much variation in scores. As such, both hypotheses are supported. That is to say, the mPPI improved participants’ levels of well-being and hope from pre- to post-intervention, accounting for the random effects from local authorities, schools, and participants. Also, the mPPI reduced participants’ symptoms of mental distress from pre- to post-intervention, accounting for the random effects from local authorities, schools, and participants.

These results add more evidence to the idea that PPIs need not take place over long durations, as in the example of Shoshani and Steinmetz (2014), using time and resources that might otherwise be used for more academic learning, which, as discussed earlier, can often be prioritized above student mental health (Waters, 2011).

Interventions can impact on three levels—individual factors, community-related factors, and system-related factors (Fazel et al., 2014). The results here show that individual factors play a large part in the outcomes of the Hummingbird Project. Though a large contribution of community factors, namely county (local authority) and school-related factors, were not shown, a large proportion of error variance remains in the models. This is not, however, uncommon when delivering interventions in uncontrolled settings, or by way of measurement error for the key variables. Research designs which afford greater experimental control would likely yield reduced error variability (Cohen, 1988), though the implementation of these designs is limited in education settings due to limited resources and constraints on participant burden imposed by gatekeepers. Indeed, the very imposition of these limits not only points to educators’ lack of priority of student mental health, discussed above, but also that school-related factors do have a significant effect on the efficacy of PPIs, even though this has not been demonstrated in this case. It is, therefore, important that researchers attempting to implement PPIs in the school setting attempt to account for such factors. It would also be advisable to conduct further studies accounting for and attempting to measure these effects.

Although not compared statistically, compared to results from cohort 1 (Platt et al., 2020), this second year of delivery of the Hummingbird student cohort also displayed a larger improvement in well-being, but with a smaller improvement in hope. Given the delivery of the mPPI was during the early part of the pandemic, and particularly early on, when the levels of uncertainty may have been more heightened, this result remains optimistic. Children can be particularly vulnerable to the mental ill-health-related effects of uncertainty, particularly if others around them are experiencing the effects of uncertainty, too. Irrespective, an increase in well-being following the Hummingbird Project was still found. Also, while there was a smaller improvement in hope than those who participated in the first year of delivery (which may also be explained by the heightened levels of uncertainty), a significant increase in hope was still found, too. It is important to note, however, that the children who participated in the second year of delivery were different from those who participated in the first year and, as such, some effects of individual differences may be at play here.

With the dosage of the intervention being relatively small, associated small effects are promising. Larger doses may yield larger effects, although this would require further testing. It would also likely be difficult to implement due to the logistical constraints of testing in schools. It is also likely that, as the intervention facilitator did not benefit from having a pre-existing relationship with the students (Paulus et al., 2016), greater effects could be observed should Hummingbird be delivered by a class teacher. Stockings et al. (2016) have shown teacher delivery to be more effective than clinician delivery for internalizing disorders. Although a recommendation for teacher training, as teachers seldom receive mental health training (Byrne et al., 2015) and report feeling uncomfortable intervening in student well-being issues for fear of making things worse (Hatton et al., 2017), the use of a facilitator may, therefore, be a necessity for brief mPPIs. It is also worth noting that facilitated interventions give students the time and opportunity to express feelings and emotions. They might also present teachers with the opportunity to see and hear what might be helpful for individual students. Hence, offering training to teachers so they can carry out the intervention within the classroom could be of interest to institutions as a way to incorporate the benefits of the interventions into the whole school setting. If teachers are finding benefit from the interventions, they are also more likely to be able to communicate this with enthusiasm to their students.

It stands to reason that improvements in levels of hope (CHS) would be found in participants in the project, not least because this is one of the concepts covered in the intervention itself. However, improvements in mental distress resulting from an intervention that does not address this construct may at first seem counterintuitive. Promoting positive mental health can ‘buffer’ the negative effects of mental illness (Trompetter et al., 2017) and produce health and social benefits (Lyubomirsky et al., 2005; Keyes, 2007). Therefore, an intervention that improves mental health can be expected to lead to improvements in educational attainment, productivity, longevity, and reduced risk of both suicide and mental illness (Campion et al., 2012). Indeed, an intervention that leads to flourishing can be expected to reduce languishing (i.e., low levels of satisfaction, purpose, meaning and fulfillment; Keyes and Haidt, 2010), as has been shown in the case of the Hummingbird Project.

### Limitations

On the one hand, a limitation that could be raised is that the effect sizes were overall small. However, in response to this, the observed effect sizes for the present cohort of participants are within the range to be expected for universal prevention programs of this type delivered to school-aged children of 0.07–0.16 (Tanner-Smith et al., 2018), with the estimates (marginal *R*^2^) when converted to Cohen’s *d* (Cohen, 1988) ranging from 0.14 to 0.20. While likely not perceptible at the individual level, they can also be meaningful at the population level (Cohen, 1988; Kraemer and Kupfer, 2006) in the context of prevention. The reporting of small effects following PPIs is also quite common (White et al., 2019), with even small changes in a positive direction promising, particularly for students at risk (DeBiase et al., 2022).

The two time points of the intervention do not allow for the inclusion of varying slopes in the models. That is to say, one data point more than cells per individual (i.e., three data points for random intercept and random slopes) is required. As such, it is impossible to estimate the varying rate of improvement, and the estimates are for the sample as a whole. It is also likely that rates of improvement are not fixed and differ at an individual level. Future studies, therefore, may want to increase the number of time points to allow for this more refined estimation and to measure any potential stability of change found between pre- and post-intervention.

Additionally, the lack of control groups in this study makes it impossible to state with certainty that the observed effect was due to the intervention, or possibly a placebo effect. Furthermore, the lack of a control group makes it possible that the effects are in part due to regression to the mean, or possibly by unaccounted-for tertiary variables. Thus, describing the intervention as a causal factor for the salutary effects observed would be beyond the scope of the data. Studies that included a control group would be more able to speak to the causes of these observed improvements.

That said, when designing such a study, one must consider the difficulties of recruitment in a school context. Resources and time are limited (Crawford and Keynes, 2015), which calls for pragmatic decisions. This was the case for the Hummingbird Project. Schools recruited into the study could not allocate more time to the study than was given, as participant burden for three-time points was judged too high and control groups were not deemed acceptable by gatekeepers. This remains a limitation, and replication with robust designs is necessary to strengthen the evidence base for this mPPI.

Data for the well-being outcome (WHO-5) replicate previously published findings (Platt et al., 2020) with effect sizes being comparable (i.e., small effects). However, while the effect of the Children’s Hope Scale did replicate, it was not of the same magnitude in the current study, being smaller. As described previously, it is possible that the national context impacted the outcomes of the study, but the lack of consistency may simply indicate that the variation in effect sizes is due to a cohort effect. Alternatively, the intervention delivery (facilitated by the lead author, IP) may have improved in line with the growing experience of the facilitator (Coetzee et al., 2021). The YP-CORE, measuring psychological distress, was an additional inclusion for the second year of the Hummingbird project; thus, it is a novel finding which will require replication.

Although the impact of the lockdowns on the outcomes of the study cannot be clearly established, they did prematurely end the delivery of the intervention. Schools where delivery was impacted by the lockdown were omitted from the analyses, as these participants were unable to complete post-intervention measures. The potential for future school closures due to local or national contexts does suggest that in order for PPIs to reach those in need when they need it most, delivery must be flexible. Therefore, we recommend that the feasibility of an online version of the Hummingbird Project should be assessed. Comparing self-guided (completed at participant’s own pace following pre-prepared materials) against facilitated (completed with a facilitator at a mutually convenient time) online interventions would allow the comparison of the effectiveness of varying modes of delivery. An online facilitated intervention also has the potential to be more effective than a self-guided online intervention (Fischer et al., 2020), and it may even outperform the school-based version, given the typically small effect sizes shown in such a setting. However, this is an empirical question, and data obtained from such a comparison would inform decisions where resources are limited, by indicating if the relative gains in effectiveness are worth the resource cost.

The YP-CORE was used to measure symptoms of mental distress in this study and, compared to the effects of the Hummingbird Project on hope and well-being, its’ effects on symptoms of mental distress showed the smallest effect. Interestingly, however, the YP-CORE also had the smallest standard deviations. This suggests that participants displayed low distress symptomology and, as such, this might be an indication of restriction of range in scores. That is, the effects of the mPPI were subdued due to participants being clustered around low distress symptomology scores; higher scores, by and large, were not present. This is a limitation and yet also a possible strength. Even though standard deviations were small, a small effect of the mPPI was found. If participants had higher levels of distress, a larger effect size might have been found. The standard deviations for hope and well-being did have larger standard deviations and, as such, less restriction of range. Although also an issue in younger children, 50% of lifetime mental illness starts by age 14 and increases to 75% by the mid-20s (Kessler et al., 2007). The majority of participants in this study were in Year 7 (age 11–12 years). As such, future research needs to expand to older age groups to see if the benefits of the Hummingbird Project on mental distress increase with increasing age. By implementing a longer-term evaluation of the mPPI, the potential of the Hummingbird Project to ‘buffer’ against the development of mental distress most often associated with older-aged secondary school students could also be explored. As no follow-up data are currently available, there is no option to explore this yet.

## Conclusion

The findings of the current study concur with, and expand upon, previous research (Platt et al., 2020) by demonstrating small improvements in well-being and hope, together with a small but significant reduction in distress from pre- to post-intervention. Since mental ill-health has higher costs for schools (Fazel et al., 2014), an mPPI which benefits both mental health and mental distress will be of more interest. The effectiveness of this mPPI has now also been demonstrated with regard to well-being and hope in two consecutive cohorts, which provides incremental support for the effectiveness of the Hummingbird Project mPPI. However, caution must be observed in interpreting these results, as causal inferences are not possible due to the pragmatic restraints which limited the study design. As such, and particularly given the replication crisis more generally, continued replication efforts are warranted to further the evidence base for this promising prevention program with more robust evaluation design.

## Data availability statement

The raw data supporting the conclusions of this article will be made available by the authors on request, without undue reservation.

## Ethics statement

The studies involving humans were approved by the Departmental Research Ethics Committee, Psychology Department, University of Bolton. The studies were conducted in accordance with the local legislation and institutional requirements. Written informed consent for participation in this study was provided by the participants’ legal guardians/next of kin.

## Author contributions

IP: Methodology, Project administration, Resources, Writing – original draft. KH: Formal analysis, Funding acquisition, Software, Writing – original draft. MT: Conceptualization, Funding acquisition, Supervision, Writing – review & editing. CK: Supervision, Writing – review & editing. JC: Conceptualization, Supervision, Writing – review & editing. CM: Writing – review & editing. CN: Conceptualization, Funding acquisition, Project administration, Resources, Supervision, Writing – review & editing.
